# [^18^F]FAPI adds value to [^18^F]FDG PET/CT for diagnosing lymph node metastases in stage I-IIIA non-small cell lung cancer: a prospective study

**DOI:** 10.1186/s40644-024-00701-y

**Published:** 2024-06-03

**Authors:** Youcai Li, Yin Zhang, Zhihua Guo, Peng Hou, Jie Lv, Miao Ke, Shaoyu Liu, Siwen Li, Weiqiang Yin, Jianxing He, Xinlu Wang

**Affiliations:** 1 Department of Nuclear Medicine, The First Affiliated Hospital of Guangzhou Medical University, Guangzhou, 510000 China; 2 Nanfang PET Center, Southern Medical University Nanfang Hospital, Guangzhou, Guangdong Province China; 3 Department of Thoracic Surgery, Guangzhou Institute of Respiratory Health & China State Key Laboratory of Respiratory Disease, The First Affiliated Hospital of Guangzhou Medical University, National Clinical Research Center for Respiratory Disease, Guangzhou, China; 4 Guangzhou Medical University, Guangzhou, China

**Keywords:** Non-small cell lung cancer, FAPI, FDG, Lymph node metastases

## Abstract

**Background:**

This study investigates the value of fluorine 18 ([^18^F])-labeled fibroblast activation protein inhibitor (FAPI) for lymph node (LN) metastases in patients with stage I-IIIA non-small cell lung cancer (NSCLC).

**Methods:**

From November 2021 to October 2022, 53 patients with stage I-IIIA NSCLC who underwent radical resection were prospectively included. [^18^F]-fluorodeoxyglucose (FDG) and [^18^F]FAPI examinations were performed within one week. LN staging was validated using surgical and pathological findings. [^18^F]FDG and [^18^F]FAPI uptake was compared using the Wilcoxon signed-ranks test. Furthermore, the diagnostic value of nodal groups was investigated.

**Results:**

In 53 patients (median age, 64 years, range: 31–76 years), the specificity of [^18^F]FAPI for detecting LN metastasis was significantly higher than that of [^18^F]FDG (*P* < 0.001). High LN risk category, greater LN short-axis dimension(≥ 1.0 cm), absence of LN calcification or high-attenuation, and higher LN FDG SUV_max_ (≥ 10.1) were risk factors for LN metastasis(*P* < 0.05). The concurrence of these four risk factors accurately predicted LN metastases (Positive Predictive Value [PPV] 100%), whereas the presence of one to three risk factors was unable to accurately discriminate the nature of LNs (PPV 21.7%). Adding [^18^F]FAPI in this circumstance improved the diagnostic value. LNs with an [^18^F]FAPI SUV_max_<6.2 were diagnosed as benign (Negative Predictive Value 93.8%), and LNs with an [^18^F]FAPI SUV_max_≥6.2 without calcification or high-attenuation were diagnosed as LN metastasis (PPV 87.5%). Ultimately, the integration of [^18^F]FDG and [^18^F]FAPI PET/CT resulted in the highest accuracy for N stage (83.0%) and clinical decision revisions for 29 patients.

**Conclusion:**

In patients with stage I-IIIA NSCLC, [^18^F]FAPI contributed additional valuable information to reduce LN diagnostic uncertainties after [^18^F]FDG PET/CT. Integrating [^18^F]FDG and [^18^F]FAPI PET/CT resulted in more precise clinical decisions.

**Trial registration:**

The Chinese Clinical Trial Registry: ChiCTR2100044944 (Registered: 1 April 2021, https://www.chictr.org.cn/showprojEN.html?proj=123995*).*

## Introduction

Lung cancer is known as the most common tumor with the highest morbidity and mortality worldwide [1]. Approximately 80% of lung cancers are non-small cell lung cancer (NSCLC) [2]. Precise tumor staging is important for effective therapy. Currently, surgery remains the primary treatment for stage I-IIIA NSCLC [3, 4]. Lymph node (LN) metastasis is an important factor affecting the prognosis of NSCLC patients, since thorough dissection of metastatic LNs plays a crucial role in improving overall survival and disease-free survival [5]. Thus, LN metastases must be accurately assessed prior to surgery.

Fluorine-18 fluorodeoxyglucose positron-emission tomography/computed tomography ([^18^F]FDG PET/CT) is superior to other imaging modalities in nodal staging because it provides metabolic information beyond morphological features [6]. As the most sensitive imaging tool for nodal staging of lung cancer for now, [^18^F]FDG PET/CT is used for preoperative lung cancer assessment [7]. However, false-positive results from [^18^F]FDG PET/CT are common problems in the nodal staging of lung cancer [8], especially in individuals with chronic lung disease. These diseases are often accompanied by reactive LN changes (inflammatory episodes or infection) that exhibit higher levels of glucose-6-phosphatase expression. Consequently, some LNs are misdiagnosed as metastases, causing patients to lose the opportunity for surgery. In addition, the resolution of [^18^F]FDG PET may still be insufficient to distinguish lymph node metastases in tiny lymph nodes and the lymph nodes with a diameter of 5 mm or less may be missed. As a result, negative [^18^F]FDG PET findings may result in the neglect of some occult lymph nodes that could have been completely removed. Therefore, improving the accuracy of imaging techniques for LN staging is critical in guiding the surgical approach.

In recent years, imaging and therapeutic research targeting fibroblast activation proteins have gained increasing attention [9, 10]. Radionuclide-labeled fibroblast activation protein inhibitor (FAPI) is a promising tumor imaging agent for lung cancer patients [10, 11]. Previous research results demonstrated that FAPI PET/CT reveals more LN lesions and provides clear delineation than [^18^F]FDG PET/CT in lung cancer patients [11, 12]. However, those suspected positive lymph nodes were not confirmed by pathology. Furthermore, prior studies have primarily focused on patients with advanced lung cancer [11–13], indicating that the role of FAPI PET/CT in detecting LN metastases in early-stage NSCLC patients requires further investigation. Additionally, gallium-68 ([^68^Ga]) FAPI has been the radiotracer in most previous studies. The low elution dose and short half-life of [^68^Ga] limit its use in clinical practice. In contrast, [^18^F] has been the most commonly used radionuclide in clinical practice due to its more abundant production, longer half-life, and higher spatial resolution. Currently, literature on FAPI primarily focuses on comparing the two imaging agents (FDG and FAPI). There is a lack of research on how to select appropriate imaging agents for lung cancer patients. The criteria for determining which patients are suitable for FDG PET/CT and which would benefit from the combination of FDG and FAPI remain inconclusive.

In this study, we aimed to investigate the value of [^18^F]FAPI PET/CT in improving the diagnostic accuracy for LN staging in patients with early-stage (I-IIIA) NSCLC, and evaluate the role of [^18^F]FAPI in helping surgeons to make optimized clinical decisions, using surgical and histological results as the gold standard.

## Materials and methods

### Patients

**Fig. 1:**
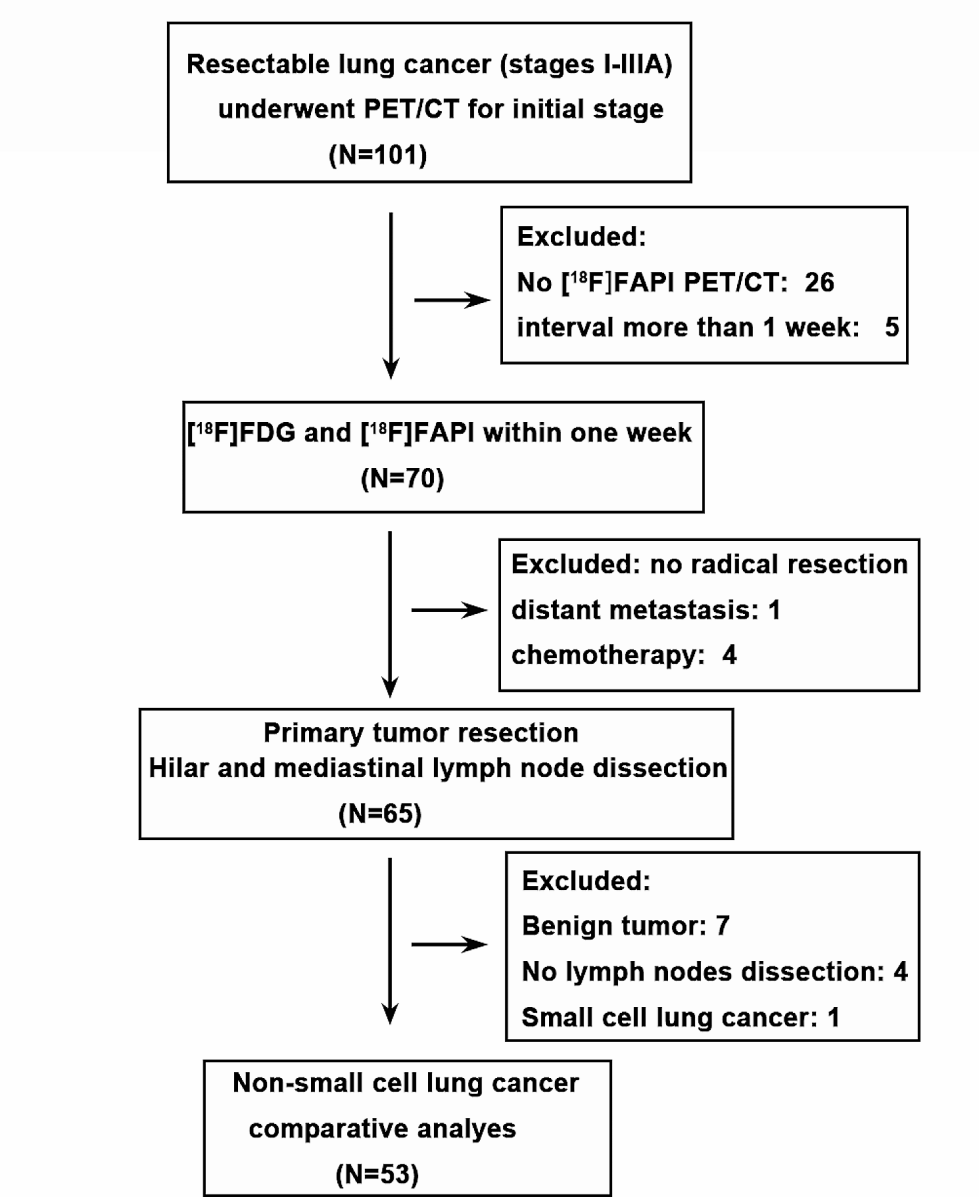
The study flowchart

This single-center prospective study was approved by the Clinical Research Ethics Committee of the First Affiliated Hospital of Guangzhou Medical University and registered with the Chinese Clinical Trial Registry (http://www.chictr.org.cn, number ChiCTR2100044944). Each patient provided informed consent. From November 2021 to October 2022, 53 patients (31 males and 22 females, median age 64 years, IQR 56–69 years old) at the First Affiliated Hospital of Guangzhou Medical University were recruited according to the following inclusion criteria: (1) patients with stage I-IIIA NSCLC; (2) patients who underwent [^18^F]FDG and [^18^F]FAPI PET/CT for initial staging within one week; and (3) patients who underwent radical resection and hilar and mediastinal lymph node dissection within 40 days after PET/CT imaging. The exclusion criteria included: (1) patients who underwent tumor-related therapy before PET/CT or surgery; (2) patients with a previous tumor history. The study flow diagram is shown in Fig. 1. The Eighth Edition of the International Association for the Study of Lung Cancer (IASLC) TNM classification of NSCLC was utilized for staging [4]. Histopathology results from resected surgical specimens or aspiration biopsy were used as the gold standard.

### Synthesis of [^18^F]FDG and [^18^F]FAPI

**Fig. 2:**
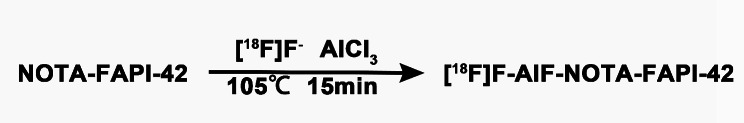
The synthesis formula of [^18^F]F-AlF-NOTA-FAPI-42

[^18^F]FDG was manufactured in accordance with the standard method [14] described by our laboratory, using the Tracer lab F_X_FDG synthesis system (GE Healthcare). The FAPI precursor (NOTA-FAPI-42) was manufactured by CSBio (Shanghai). The synthesis of [^18^F]F-AlF-NOTA-FAPI-42 (Abbreviated as [^18^F]FAPI in the following text) is shown in Fig. 2. Radiochemical purity was over 95% for both [^18^F]FDG and [^18^F]FAPI. The final products were diluted with saline and sterilized by filtering with a 0.22-μm Millipore filter (Millipore Sigma, Burlington, MA) into a sterile multidose syringe. The final product was tested as sterile and pyrogen free.

### [^18^F]FDG and [^18^F]FAPI PET/CT acquisition

All PET/CT studies were carried out using a PET/CT scanner (Discovery MI; GE Healthcare, WI). There was no fixed sequence for [^18^F]FDG and [^18^F]FAPI examinations. Before receiving an [^18^F]FDG injection, patients were required to fast for at least six hours, ensuring all patients had blood glucose levels below 7.0 mmol/L. Neither fasting nor normal blood glucose levels were required for the [^18^F]FAPI PET/CT examination. We carried out a PET/CT scan from the head to the mid-thigh at approximately 60 min (range: 50 to 90 min) after intravenous injection of [^18^F]FAPI (3.7 MBq/kg) or [^18^F]FDG (3.7 MBq/kg). All PET/CT studies were carried out using a PET/CT scanner (Discovery MI; GE Healthcare, WI). After the whole body PET/CT scan, patients underwent thin-slice CT chest scan while holding their breaths. Data obtained by Discovery MI were transferred to the Advantage Workstation (version AW 4.7; GE Healthcare) and were reconstructed by using the Bayesian penalized likelihood reconstruction algorithm (Q.clear; GE Healthcare) with a penalization factor (b) of 750. The reconstructed images were then co-registered and displayed. The median time interval between [^18^F]FDG and [^18^F]FAPI PET/CT was two days (IQR 1–4 days).

### Imaging analysis

Four experienced nuclear medicine specialists were divided into two groups to interpret the PET/CT imaging (FDG: Y.Z. and P.H., 9 and 12 years of experience, respectively; FAPI: Y.L. and X.W., 7 and 20 years of experience, respectively). All disagreements were discussed to reach consensus. The study interpreters were blinded to clinical data and other PET/CT information during PET/CT interpretation. On PET (FDG or FAPI) scans, the area with focal tracer uptake higher than the background (mediastinum blood pool) was considered to be positive [9, 12]. The maximum standard uptake value (SUV_max_) was calculated for primary tumors and LNs.

According to the IASLC LN map criteria for lung cancer staging [15], nodal groups were evaluated and allocated to eleven groups (Group 1, supraclavicular zone (1R, right; 1 L, left); Group 2, upper paratracheal lymph node (2R, right; 2 L, left); Group 3, prevascular and retrotracheal lymph node; Group 4, lower paratracheal lymph node (4R, right; 4 L, left); Group 5, subaortic lymph node; Group 6, paraaortic lymph node; Group 7,subcarinal lymph node; Group 8, paraesophageal lymph node (8R, right; 8 L, left); Group 9, pulmonary ligament lymph node; Group 10, hilar lymph node (10R, right; 10 L, left); Group 11: interlobar lymph nodes (11R, right; 11 L, left)). Simultaneously, all LNs were classified into high-, moderate-, or low-risk categories based on LN and lung nodule location [5, 13]. The LN risk category was listed in Table 1. In addition, calcification (CT value more than 200Hu) or high-attenuation (CT value greater than 70Hu or greater than the major mediastinal arteries) was also evaluated for LN [16]. In this paper, calcification or high-attenuation is abbreviated as CHA.

**Table 1:** A list of lymph node risk categories

Lung nodule location	High-Risk	Intermediate-Risk	Low-Risk
Left upper lobe	10–11 L, 4 L,5,6	1 L,2 L,3,7,8,9	1R,2R,4R,10R
Left lower lobe	10–11 L, 4 L,7	1 L,2 L,3,5,6,8,9	1R,2R,4R,10R
Right upper lobe	10-11R, 4R	1R,2R,3,7,8,9	1 L,2 L,4 L,5,6,10 L
Right middle lobe	10-11R, 4R,7	1R,2R,3,8,9	1 L,2 L,4 L,5,6,10 L
Right lower lobe	10-11R, 4R,7	1R,2R,3,8,9	1 L,2 L,4 L,5,6,10 L

### Surgical and histopathologic analysis

Patients with clinically estimated N1-2 stage were eligible for surgery. An experienced surgical team (Z.G. and W.Y., 12 and 31 years of experience, respectively) removed the tumor and dissected LNs via thoracoscope after a systemic preoperative evaluation. In our surgical procedure, the surgeons sampled all visible and palpable LNs that were accessible in the ipsilateral hilum and mediastinum [16]. All encountered lymph nodes were removed from the IASLC lymph node map areas of 10R, 9R, 8R, 7, 4R, 3, and 2R in tumors of the right lung and from areas 10 L, 9 L, 8 L, 7, 6, 5, and 4 L of the left lung [16]. When necessary, particularly if imaging indicated a positive lymph node in non-routine dissection groups (e.g., group 1 for highest mediastinal or 2 L for tumors in the left lung), such groups were also assessed during mediastinoscopy. For N staging of NSCLC, surgeons labeled the dissected LNs by nodal groups according to the LN map criteria. The tumors and LNs (location and number) were evaluated by a lung pathologist with a decade of experience. The pathologic stage of each participant was recorded.

If contralateral hilar or mediastinal LNs, ipsilateral or contralateral scalene nodes, and supraclavicular LNs were interpreted as positive on [^18^F]FDG or [^18^F]FAPI PET/CT, the patients were considered to have suspicion of N3 stage. These LNs of N3 stage were further evaluated by the surgeon using needle biopsy.

### Statistical analysis

IBM SPSS Statistics software (version 24) was used to conduct the statistical tests. [^18^F]FDG and [^18^F]FAPI uptake was compared using the Wilcoxon signed-ranks test. Analysis of the influence of parameters on the differential diagnosis of metastatic and nonmetastatic LNs was conducted using the chi-square test and logistic regression. The area under the receiver operating characteristic (ROC) curve was used to analyze the predictive value of [^18^F]FAPI SUV_max_ and [^18^F]FDG SUV_max_. Diagnostic performance was assessed. McNemar tests were used to calculate marginal differences in sensitivity, specificity and accuracy between [^18^F]FDG and [^18^F]FAPI. The interobserver agreement was calculated using k-statistics. We considered statistically significant differences at *P* < 0.05.

## Results

### Patient characteristics

A total of 242 nodal groups from 53 patients (31 males and 22 females, median age 64 years, IQR 56–69 years old) were evaluated. The patient characteristics are shown in Table 2. All N1-N2 LNs (210 nodal groups) were verified by surgical pathology. In 19 patients,32 nodal groups were interpreted as N3 stage on [^18^F]FDG or [^18^F]FAPI PET/CT scans, but biopsies proved these LNs to be benign. The [^18^F]FDG based (kappa = 0.85) and [^18^F]FAPI based (kappa = 0.78) interobserver agreements for the diagnosis of LN were high.

**Table 2:** Patient characteristics

Characteristics	Number
**Total patient**	53
**Median age (**IQR)	64(56–69)
**Sex**	
Male	31
Female	22
**Smoking history**	
Non-smoker	36
Smoker	17
**Histology**	
Adenocarcinoma	43
Squamous cell carcinoma	6
Otherwise specified	4
**Pathological TNM stage**	
I	27
II	12
IIIA	14
**Pathological N stage**	
N0	32
N1	8
N2	13
**Days between FDG and FAPI PET/CT** (median, IQR)	2.0(1–4)
**Days between PET/CT and surgery** (median, IQR)	7(4–14)

Abbreviation: IQR = interquartile range, FDG = fluorodeoxyglucose, FAPI = fibroblast activation protein inhibitor, PET/CT = positron-emission tomography/computed tomography

The signal behavior of primary tumors is depicted in Table 3. The median SUV_max_ for the uptake of primary lesions was measured at 10.88 for [^18^F]FAPI PET/CT and 11.17 for [^18^F]FDG PET/CT, with no significant differences between the two modalities. Additionally, the ratio of primary lesion uptake to lymph node (LN) uptake was calculated as 4.10 (median: 4.10, range: 0.42–19.85) for [^18^F]FAPI PET/CT and 2.62 (median: 2.62, range: 0.05–24.04) for [^18^F]FDG PET/CT.

**Table 3:** Comparison of [^18^F]FDG and [^18^F]FAPI uptake in primary tumors

Variables	No. of Lesions	Tumor Size Median (Range)	Median FAPI Uptake Median SUV_max_ (Range)	Median FDG Uptake Median SUV_max_ (Range)	*P* Value
**Primary tumor**	53	2.6 (1.0-5.5)	10.88(2.18–24.48)	11.17(0.88–35.07)	0.677
**T stage**					
T1	30	2.25(1.0–3.0)	10.72(2.18–23.74)	9.32(1.17–24.10)	0.967
T2-T3	23	3.4(1.5–5.5)	12.69(3.46–24.48)	13.73(0.88–35.07)	0.484
**Histology**					
ADC	44	2.6(1.0-5.5)	11.48(2.18–24.48)	9.71(0.88–35.07)	0.510
SCC	5	3.7(1.6–4.5)	12.39(8.29–20.18)	17.3(9.24–25.12)	0.043
Other	4	3.8(1.5–4.5)	10.28(2.59–52.18)	20.76(4.13–34.44)	0.715

Abbreviation: ADC = Adenocarcinoma, SCC = Squamous cell carcinoma

### Nodal group-based diagnostic value of [^18^F]FDG and [^18^F]FAPI

There were 45 nodal groups of 21 patients with pathologically confirmed metastases. Although the sensitivity of [^18^F]FAPI in nodal groups was lower than that of [^18^F]FDG PET/CT (All nodal groups: 80.0% vs. 88.9%, *P* = 0.125; N1: 76.2% vs. 90.5%, *P* = 0.25; N2: 83.3% vs. 87.5%, *P* = 1.00), the difference was not statistically significant. However, the specificity of [^18^F]FAPI was significantly higher than that of [^18^F]FDG (All nodal groups: 83.2% vs. 48.7%, *P* < 0.001; N1: 84.1% vs. 36.4%, *P* < 0.001; N2: 87.6% vs. 64.5%, *P* < 0.001), indicating that [^18^F]FDG resulted in more false-positive cases than [^18^F]FAPI PET/CT. Finally, the accuracy of [^18^F]FAPI in the diagnosis of metastatic LNs was superior to that of [^18^F]FDG (All nodal groups: 82.6% vs. 56.2%, *P* < 0.001; N1: 81.5% vs. 53.8%, *P* < 0.001; N2: 86.9% vs. 68.3%, *P* < 0.001). The results of diagnostic efficacy are shown in Table 4.

**Table 4:** Diagnostic value of [^18^F]FAPI and [^18^F]FDG PET/CT in nodal groups

Nodal group	Sensitivity (95CI%)	Specificity (95CI%)	Negative Predictive Value (95CI%)	Positive Predictive Value (95CI%)	Accuracy (95CI%)
All					
FAPI	80.0 (65.4–90.4)	83.2(77.3–88.2)	94.8 (91.0–97.0)	52.2(43.6–60.6)	82.6 (77.4–86.9)
FDG	88.9(76.0-96.3)	48.7 (41. 6-55.9)	95.1(89.3–97.8)	28.4(25.0–32.0)	56.2 (49.9–62.3)
**N1**					
FAPI	76.2(52.8–91. 8)	84.1(69.9–93.4)	88.1 (77.3–94.1)	69.6(52.7–82. 5)	81.5 (70.3–89.3)
FDG	90.5(69.6–98.8)	36.4(22.4–52.2)	88. 9(66.9–96.9)	40.4 (34.3–46.9)	53.8(41.9–65.4)
**N2**					
FAPI	83.3 (62.6–95.3)	87.6 (80.4–92. 9)	96.4(91.5–98.5)	57.1 (44.6–68.9)	86.9 (80.4–91.5)
FDG	87.5 (67.6–97.3)	64.5 (55.3–73.0)	96.3 (90.0-98.7)	32.8 (26. 9-39.3)	68.3(60.3–75.3)

Abbreviation: FAPI = fibroblast activation protein inhibitor, FDG = fluorodeoxyglucose, CI = confidence interval

### Risk factors for [^18^F]FDG PET/CT for LN metastasis

As shown in Table 5, high LN risk category, LN short-axis size (≥ 1.0 cm), and absence of LN CHA were more commonly found in metastatic LNs than in benign LNs (*P* < 0.05). The SUV_max_ of [^18^F]FDG was higher in metastatic LNs than in nonmetastatic LNs (median SUV_max_, 7.73 vs. 5.31; *P* = 0.001). ROC analysis indicated that an LN FDG SUVmax ≥ 10.1 was likely to indicate LN metastasis (AUC: 0.67; 95% CI: 0.57 to 0.77). Univariate analysis revealed that high LN risk category, large LN short-axis size (≥ 1.0 cm), absence of LN CHA, and higher LN FDG SUV_max_ (≥ 10.1) were the four risk factors for LN metastases. The co-occurrence of these four risk factors accurately predicted LN metastases (Positive Predictive Value [PPV] 100%). These 12 nodal groups were classified as the coexistence of 4 risk factors group. Whereas LNs with negative [^18^F]FDG PET/CT manifestation could excellently predict benign LNs (Negative Predictive Value [NPV] 95.0%), and these 101 nodal groups were classified as the [^18^F]FDG negative group.

**Table 5:** Analysis of the relationship between [^18^F]FAPI PET/CT and [^18^F]FDG PET/CT parameters with lymph node metastases

Variables	Benign (*n* = 197)	Metastases (*n* = 45)	*P*
**LN risk categories**			0.001
High	119(60.4)	39(86.7)	
Low to intermediate	78(39.6)	6(13.3)	
**LN short-axis**			< 0.001
< 1.0 cm	182(92.4)	16(35.6)	
≥ 1.0 cm	15(7.6)	29(64.4)	
**LN calcification or high attenuation**			0.001
Absent	143(72.6)	43(95.6)	
Present	54(27.4)	2(4.4)	
**FDG SUV**_**max**_(median, IQR)	5.31(4.04, 7.87)	7.73(4.81, 16.74)	0.001
**FAPI SUV**_**max**_(median, IQR)	1.96(1.42, 3.92)	9.85(6.05, 19.24)	< 0.001

Abbreviation: LN = lymph node, FAPI = fibroblast activation protein inhibitor, FDG = fluorodeoxyglucose, SUV_max_= the maximum standard uptake value, IQR = interquartile range

### LNs with one to three risk factors need further [^18^F]FAPI PET/CT

**Fig. 3:**
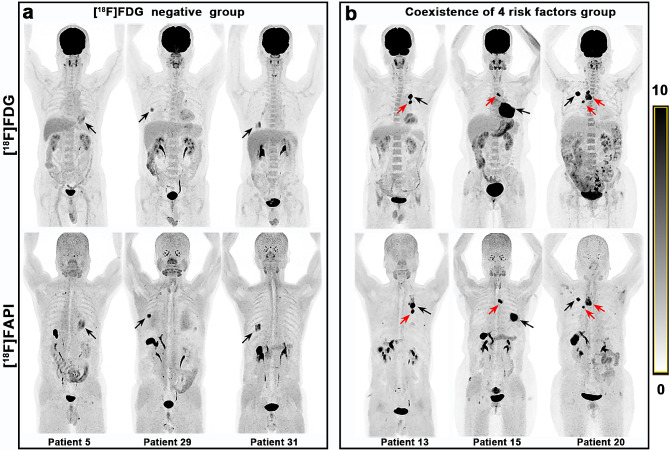
(**a**) [^18^F]FDG and [^18^F]FAPI PET/CT of 3 representative patients in the [^18^F]FDG negative group. Lymph nodes with negative [^18^F]FDG PET/CT findings were also negative on [^18^F]FAPI PET/CT. (Note: The right hilar lymph nodes in the second and third patients display slight FDG uptake, precluding their classification as [^18^F]FDG negative group, while the other lymph nodes fall into the [^18^F]FDG negative group). (**b**) [^18^F]FDG and [^18^F]FAPI PET/CT of 3 representative patients in the coexistence of 4 risk factors group. Lymph nodes indicated by the red arrows were high-risk regional lymph nodes with a diameter greater than 1.0 cm and no CHA. These lymph nodes exhibited high uptake on both [^18^F]FDG (SUV_max_=10.9–21.3) and [^18^F]FAPI (SUV_max_ =12.5–22.9) imaging. Subsequent surgical pathology confirmed these lymph nodes as metastatic (Black arrows indicated primary lesion)

**Fig. 4:**
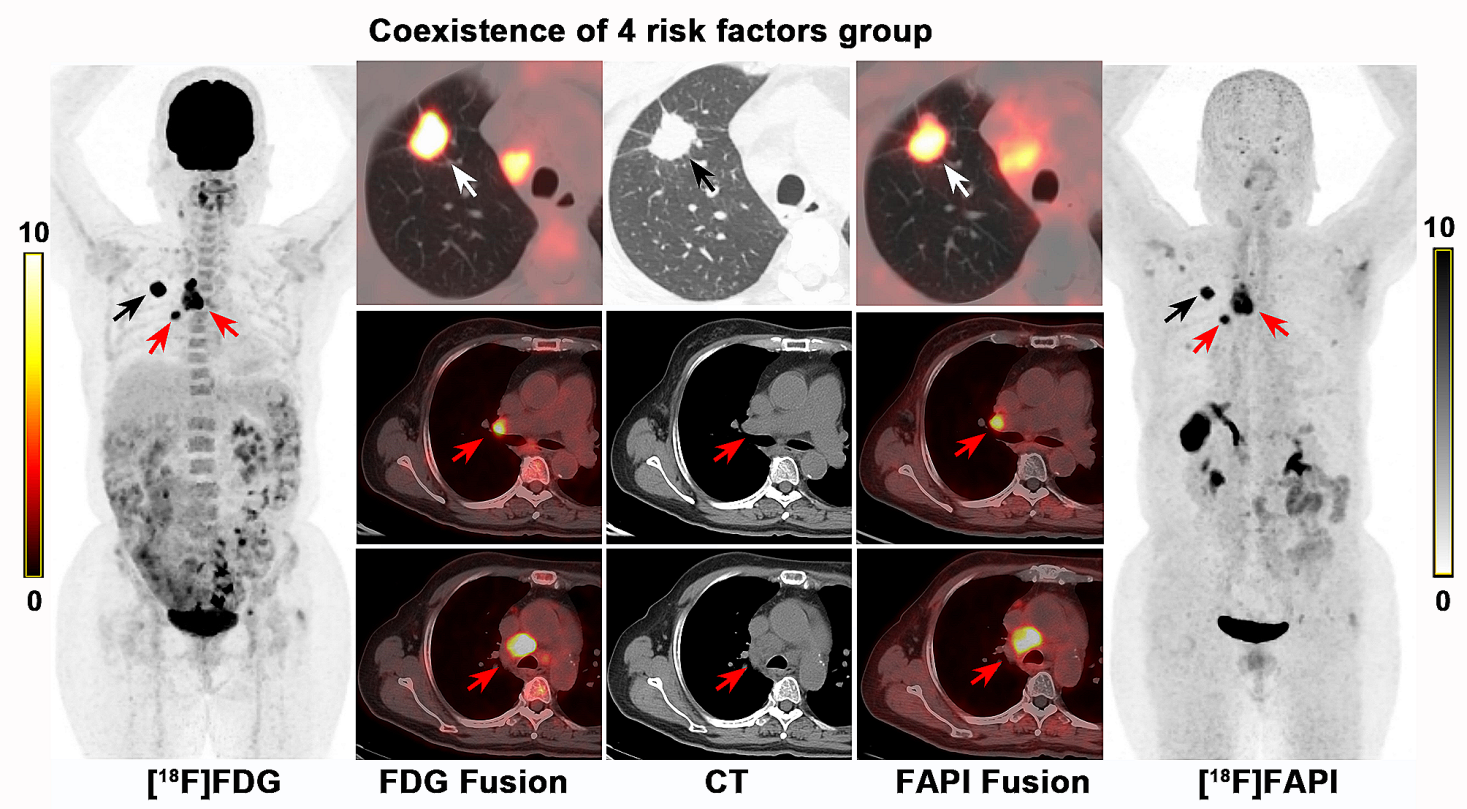
A 65-year-old woman was diagnosed as invasive adenocarcinoma of right upper lung (black or white arrow). The right hilar (10R) and mediastinal (4R) lymph nodes (red arrows) were all high-risk regional lymph nodes with a diameter greater than 1.0 cm (1.1–2.6 cm) and no CHA. These lymph nodes showed high [^18^F]FDG (SUV_max_=10.9–21.3) and [^18^F]FAPI (SUV_max_ =10.0-12.5) uptake. Surgical pathology subsequently confirmed these lymph nodes as metastatic

**Fig. 5:**
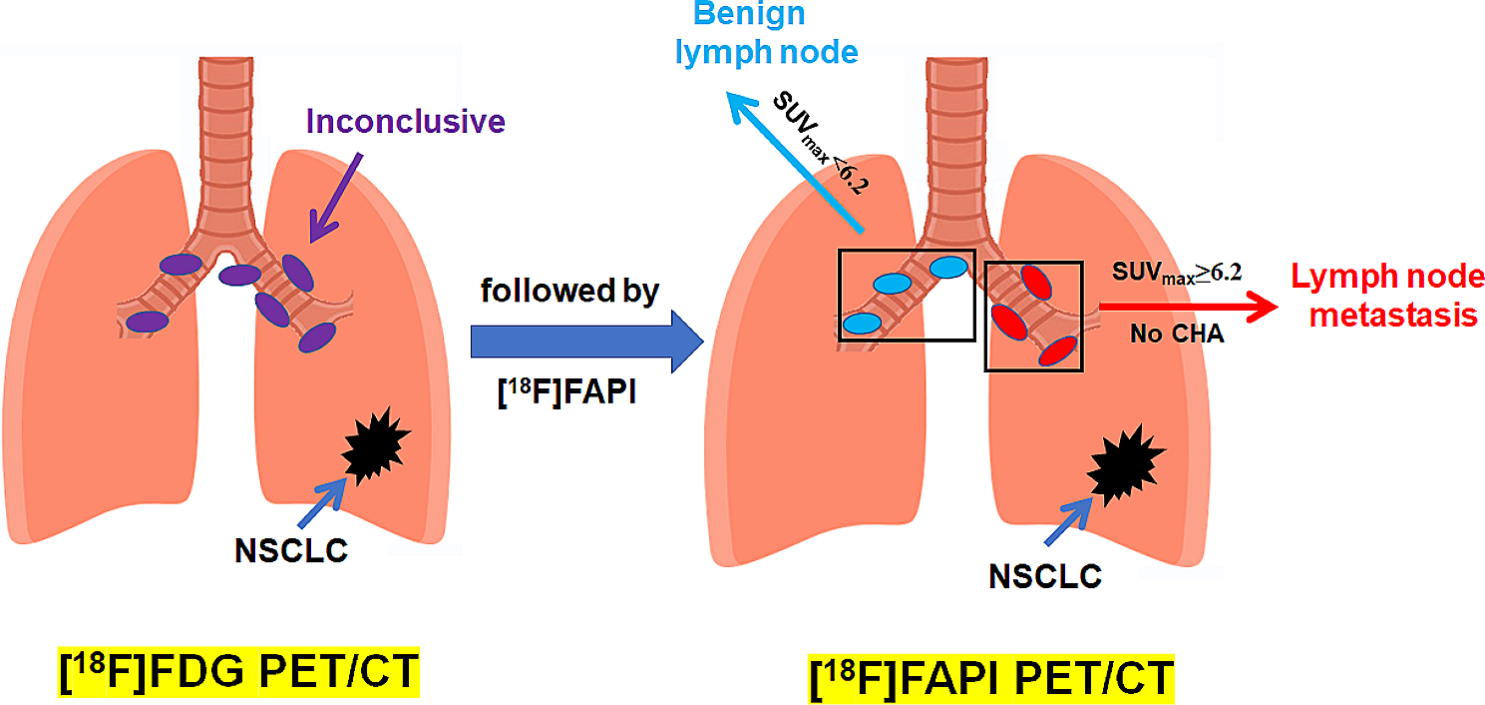
The utility of [^18^F]FAPI PET in cases of inconclusive diagnosis in nodal groups after [^18^F]FDG PET/CT examination

**Fig. 6:**
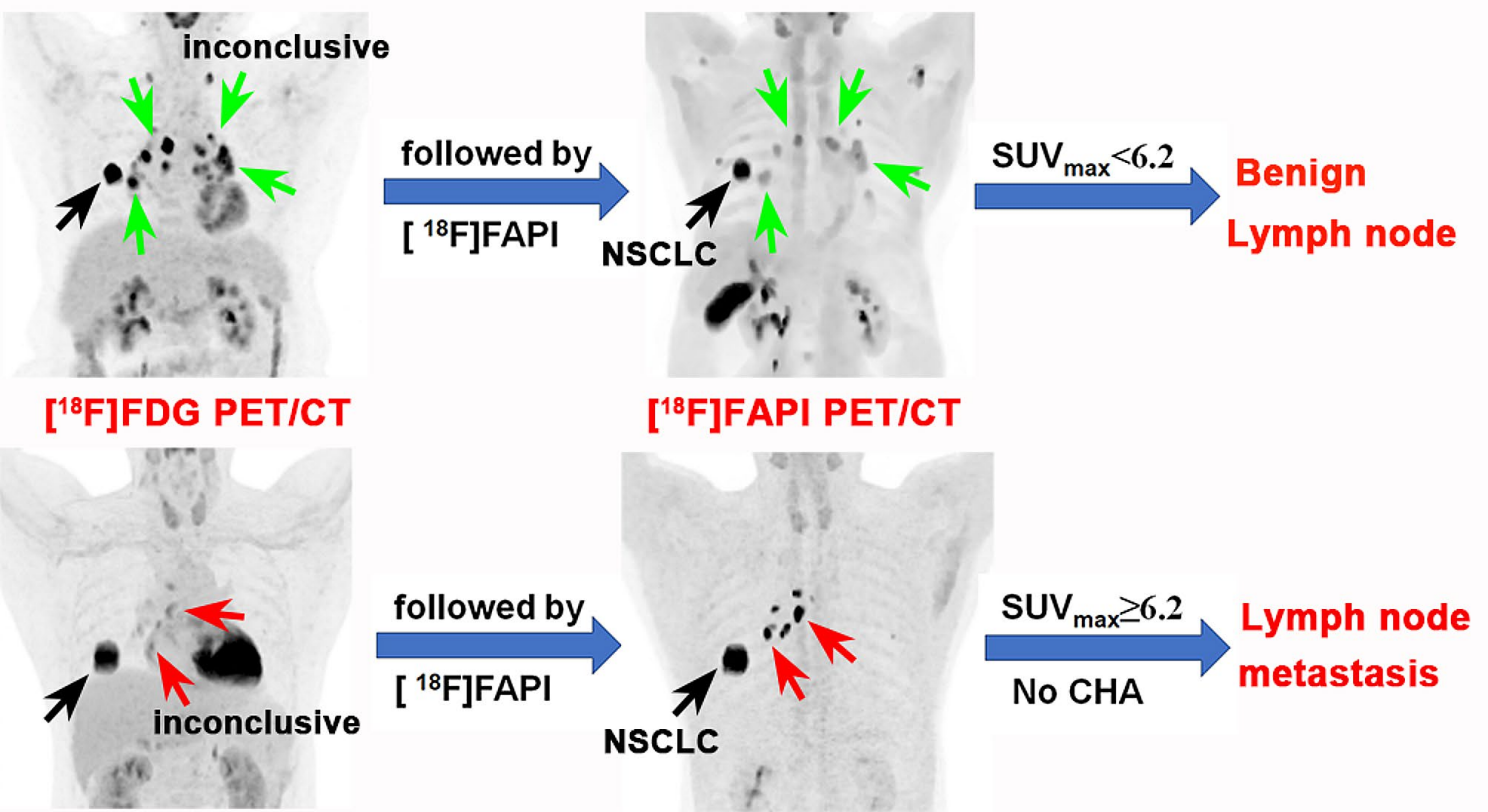
When [^18^F]FDG PET/CT was uncertain in diagnosing lymph nodes (LNs), the addition of [^18^F]FAPI provides valuable information. LNs with an [^18^F]FAPI SUVmax < 6.2 were diagnosed as benign, and LNs with an [^18^F]FAPI SUVmax ≥ 6.2 without calcification or high-attenuation were diagnosed as LN metastasis. LNs were confirmed by surgical specimens or aspiration biopsy

In the [^18^F]FDG negative group, all LNs (101 nodal groups) with negative [^18^F]FDG PET/CT findings were also negative on [^18^F]FAPI PET/CT (Fig. 3a). In the coexistence of 4 risk factors group (high LN risk category, large LN short-axis size (≥ 1.0 cm), absence of LN CHA and higher LN FDG SUV_max_ (≥ 10.1)), all LNs (12 nodal groups) diagnosed as metastases on [^18^F]FDG PET/CT also showed metastases on [^18^F]FAPI PET/CT (Figs. 3b and 4). Therefore, [^18^F]FAPI PET/CT provided no additional diagnostic value for the [^18^F]FDG negative group and the coexistence of 4 risk factors group. So, evaluating these LNs requires only an [^18^F] FDG PET/CT examination. When one to three of the four risk factors were present (classified as the 1–3 risk factor group), the diagnoses remained undetermined, further [^18^F]FAPI PET/CT were needed (Figs. 5 and 6).

### [^18^F]FAPI performs better than [^18^F]FDG in identifying LN metastases

**Fig. 7:**
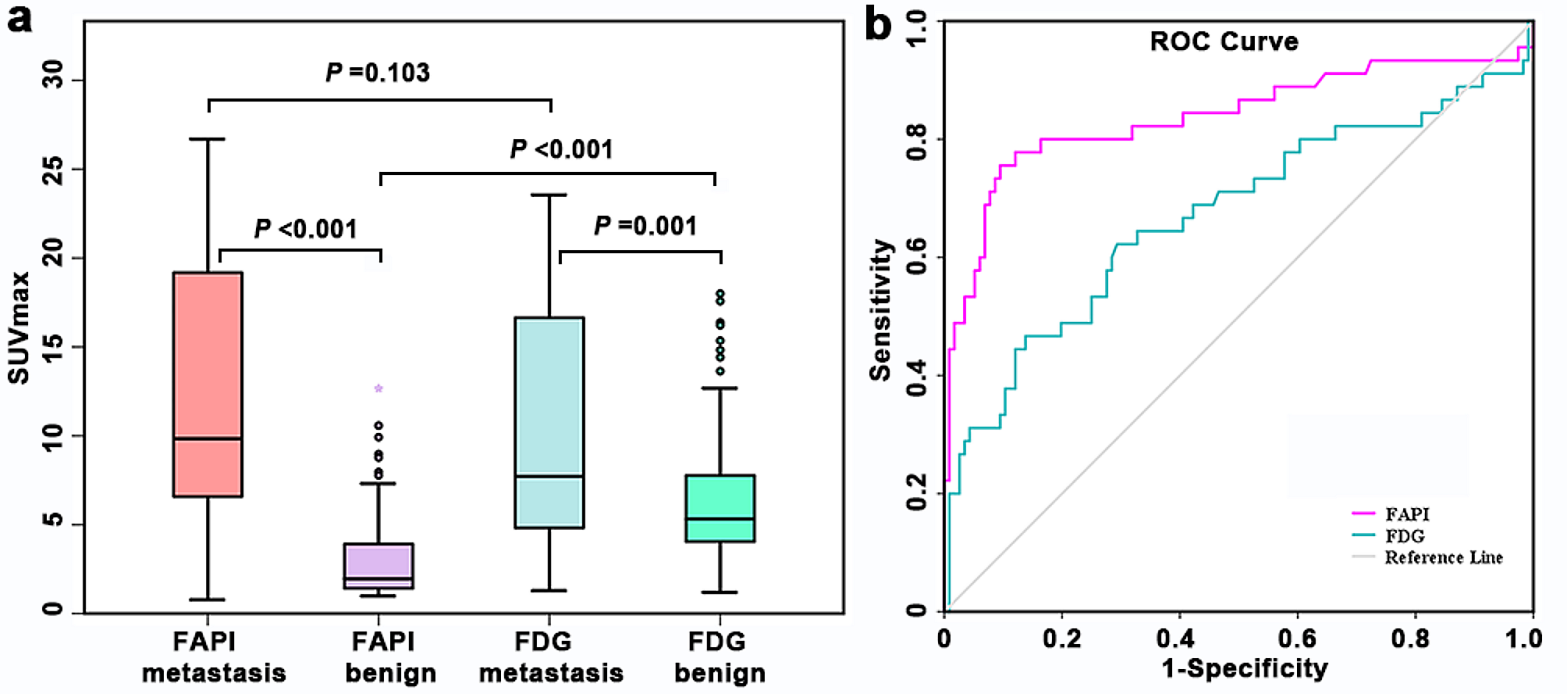
(**a**) The uptake of [^18^F]FAPI and [^18^F]FDG for benign and malignant lymph nodes. (**b**) Receiver operating curve analysis of lymph nodes SUV_max_ on FAPI or FDG

The SUV_max_ of [^18^F]FAPI was higher in metastatic LNs than in nonmetastatic LNs (median SUV_max_, 9.85 vs. 1.96; *P* < 0.001, Table 5). However, the SUV_max_ of [^18^F]FAPI was lower than that of [^18^F]FDG in nonmetastatic LNs (median SUV_max_, 1.96 vs. 5.31; *P* < 0.001). Although the [^18^F]FAPI SUV_max_ was higher than that of [^18^F]FDG in metastatic LNs, the difference was not statistically significant (median SUV_max_, 9.85 vs. 7.73; *P* = 0.103). The detail is shown in Fig. 7a. The AUC of LN [^18^F]FAPI SUV_max_ was higher than that of [^18^F]FDG SUV_max_ (0.84 vs. 0.67), indicating the better predictive value of [^18^F]FAPI in metastatic LNs (Fig. 7b). ROC analysis indicated an excellent performance of [^18^F]FAPI SUV_max_ ≥ 6.2 as an indicator of LN metastasis (AUC: 0.84, 95% CI: 0.75, 0.92; *P* < 0.001). Multivariable analysis revealed that only no LN CHA and higher LN [^18^F]FAPI SUV_max_ were associated with a higher odds ratio for LN metastasis (*P* < 0.001, Table 6).

**Table 6:** Odds ratios from multivariate analysis of predictors of lymph node metastases

Variable	Odds ratio	95% confidence interval	*P*
No LN CHA	31.27	5.06-193.42	< 0.001
FAPI SUV_max_(≥ 6.2)	105.74	31.99-349.53	< 0.001

Abbreviation: LN CHA = lymph node calcification or high-attenuation, FAPI = fibroblast activation protein inhibitor, SUV_max_= the maximum standard uptake value

### [^18^F]FAPI PET/CT contributes to reducing the diagnostic uncertainty of [^18^F]FDG PET/CT

**Fig. 8:**
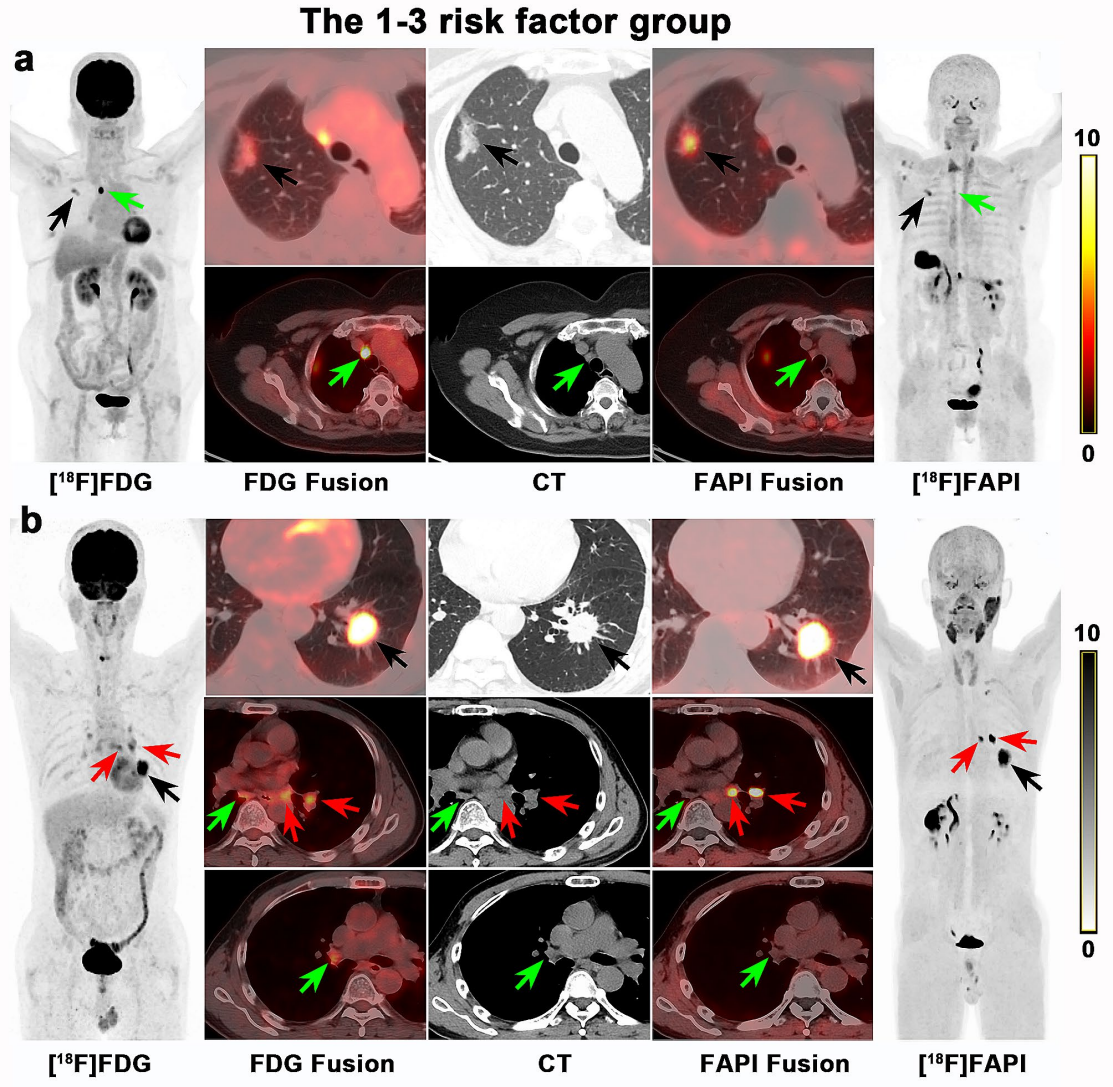
(**a**) A 70-year-old woman with right upper lung mass (black arrow) underwent PET/CT. A mediastinal lymph node (2R) with 0.8 cm in short diameter was showed high [^18^F]FDG uptake (SUV_max_ = 16.38), which was strongly suspected as metastasis. Whereas the uptake for this lymph node on [^18^F]FAPI was low (SUV_max_ = 2.37, green arrow). Finally, the lung mass was proven as invasive adenocarcinoma and this lymph node was proven as benign by surgical pathology. (**b**) A 57-year-old male was diagnosed as invasive adenocarcinoma of left lung (black arrow). The bilateral hilar (10 L,10R) and mediastinal (7, 8 L) lymph nodes showed moderate uptake on [^18^F]FDG PET/CT (SUV_max_ = 4.0-6.4). These LNs were all less than 1.0 cm (0.5–0.9 cm) in short diameter without calcification or high-attenuation. These were suspected as metastases according to the [^18^F]FDG PET/CT. On [^18^F]FAPI PET, the left hilar (10 L) and mediastinal (8 L) lymph nodes showed intensive uptake (SUV_max_ = 8.5–19.0, red arrows). Other lymph nodes showed low uptake of [^18^F]FAPI (SUV_max_ = 1.1–1.6, green arrows). Lymph nodes dissection confirmed that the Group10L and 8 L lymph nodes were metastases, and the Group 10R and 7 lymph nodes were confirmed benign lesions by biopsy

**Fig. 9:**
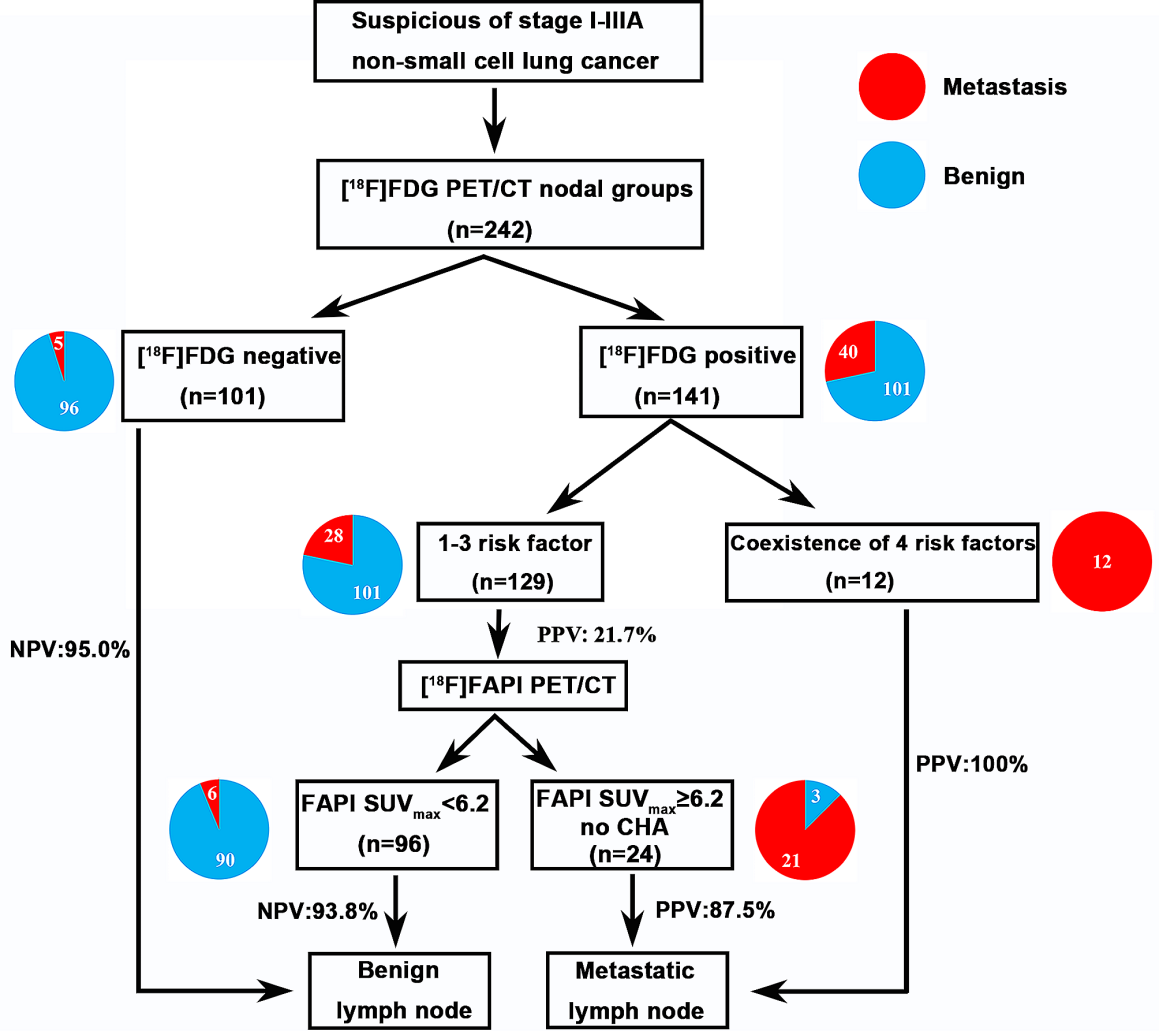
Suggested diagnostic flow for stage I-IIIA NSCLC patients in nodal groups (Note: the remaining 9 nodal groups with [^18^F]FAPI SUV_max_ greater than 6.2 and CHA were still undetermined). Abbreviation: CHA = calcification or high-attenuation, NPV = negative predictive value, PPV = positive predictive value

Among the 129 nodal groups with diagnostic uncertainty after [^18^F]FDG PET/CT (1–3 risk factor group), [^18^F]FAPI PET/CT contributed additional information (Figs. 5 and 6). Indeed, [^18^F]FAPI PET/CT correctly identified benign LNs in 90 of 96 nodal groups with an SUV_max_ lower than 6.2, which boosted the NPV to 93.8% (Figs. 8a and 9). Meanwhile, there were 24 nodal groups with an [^18^F]FAPI SUV_max_ greater than 6.2 and without LN CHA. Finally, 21 of these 24 nodal groups were proven to be metastases, and [^18^F]FAPI PET/CT boosted the PPV from 21.7 to 87.5% (Figs. 8b and 9). The remaining 9 nodal groups with an [^18^F]FAPI SUV_max_ greater than 6.2 and CHA were still undetermined. Based on these findings, we suggested a diagnostic flowchart as shown in Fig. 9.

### Changes in clinical decision-making

**Fig. 10:**
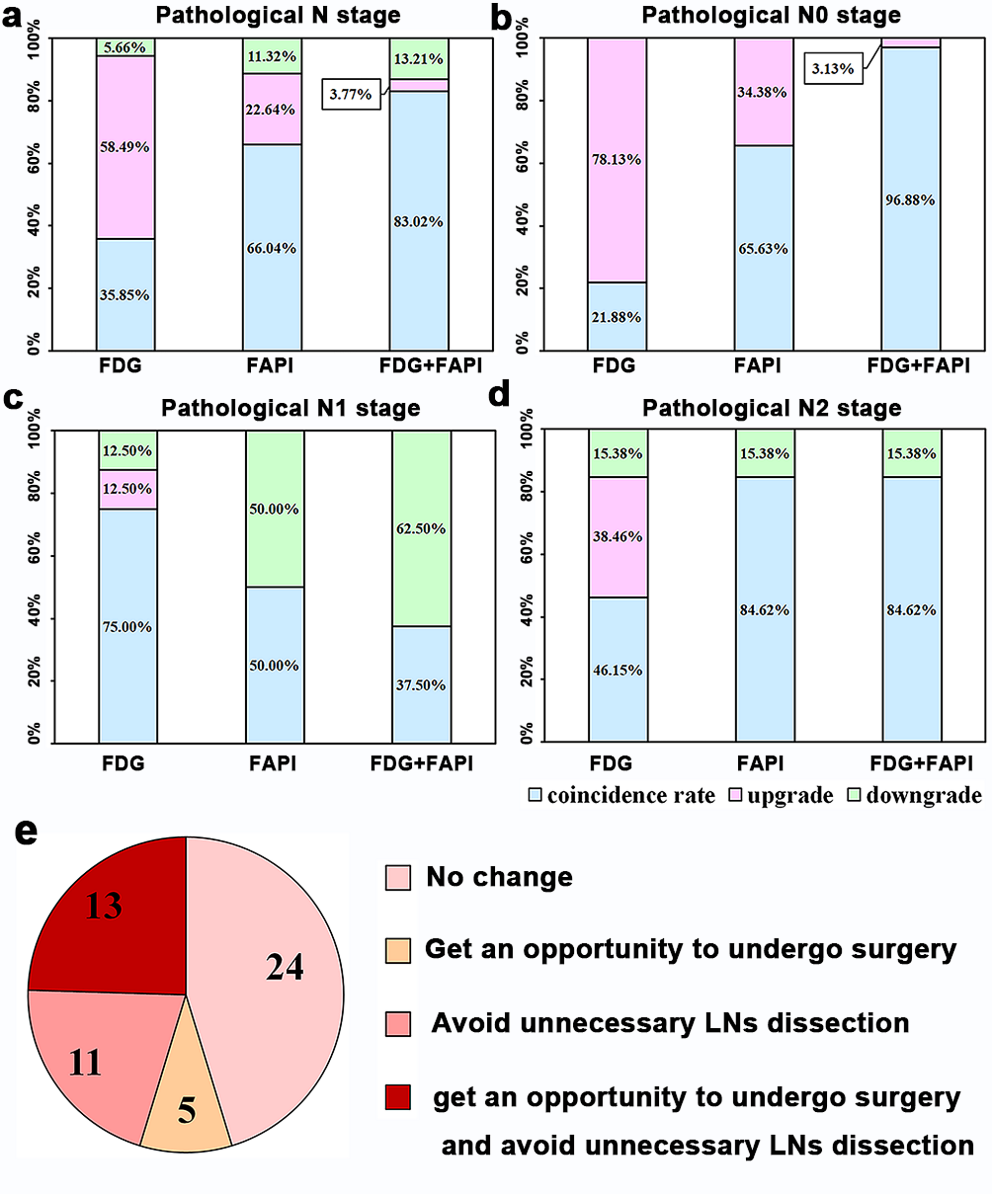
(**a**-**d**) Accuracy analysis of different types of imaging modes in N staging according to pathological results. (**e**) Compared to [^18^F]FDG PET/CT, pie chart of implemented management change after integrating [^18^F]FAPI and [^18^F]FDG PET/CT. Abbreviation: FDG + FAPI = Integrating [< Superscript>18</Superscript> F]FAPI and [< Superscript>18</Superscript> F]FDG PET/CT, lymph nodes = LNs

As shown inFigure 10a, the FDG-based and FAPI-based N staging accuracies were 35.8%, and 66.0%, respectively. Integrating [^18^F]FAPI and [^18^F]FDG (FDG + FAPI) resulted in the highest accuracy, at approximately 83.0%. Consequently, after integrating [^18^F]FAPI and [^18^F]FDG, 18 patients with N3 staging on [^18^F]FDG imaging were corrected to N0-N2 stage and were ultimately able to undergo radical resection (Fig. 10e).

False-positive LNs could result in N staging upgrades, which were more common in [^18^F]FDG PET/CT alone than in FDG + FAPI (Fig. 10), especially for pathological N0 stage. In 32 patients with N0 stage, the FDG-based N staging was overestimated in 25 (78.1%, Fig. 10b) patients. After integrating [^18^F]FDG and [^18^F]FAPI PET/CT, only one patient (3.1%) was overestimated (Fig. 10b). Consequently, 24 patients with N1-3 staging on [^18^F]FDG imaging were diagnosed with N0 after integrating [^18^F]FDG and [^18^F]FAPI PET/CT, which was later confirmed as N0 by pathology. Therefore, 24 patients could have avoided LN dissection by integrating [^18^F]FDG and [^18^F]FAPI PET/CT (Fig. 10e).

## Discussion

Our study highlights that [^18^F]FAPI adds value to [^18^F]FDG PET/CT for diagnosing lymph node metastases in stage I-IIIA non-small cell lung cancer. Among patients undergoing [^18^F]FDG PET/CT, the presence of one to three risk factors identified an equivocal diagnostic zone. For this condition, the SUV_max_ of [^18^F]FAPI provided pivotal information leading to an accurate diagnosis. LNs with an [^18^F]FAPI SUV_max_<6.2 were diagnosed as benign, while LNs with an [^18^F]FAPI SUV_max_≥6.2 without calcification or high-attenuation, were diagnosed as LN metastasis. Finally, we provide a complete diagnostic pathway for diagnosing LNs metastases.

Among patients with NSCLC, [^18^F]FDG PET/CT is widely used in preoperative staging since it is sensitive in detecting LN metastasis [17]. In several recent large trials, primary tumor location, size, histology, and SUV_max_ were not associated with LN involvement [18, 19]. Therefore, the relationship between primary tumor characteristics and LN metastases was not investigated in this study.

In this study, we discovered 4 risk factors on [^18^F]FDG PET/CT (LN risk category, CHA, LN short-axis dimension, and LN [^18^F]FDG SUV_max_) that were significantly associated with LN metastasis. Those were consistent with previous reports [5]. Patients with a high LN risk category, including ipsilateral peribronchial (Group 11), hilar (Group 10), lower paratracheal (Group 4), and subcarinal lymph nodes (Group 7), were more prone to LN metastasis. Shim et al [8] noted that nodes displaying calcification or higher attenuations than that of the surrounding great vessels, even with positive uptake at PET, are benign. These nodes show follicular hyperplasia in the cortex, anthracotic pigmentation, and macrophage infiltration with or without fibrotic micronodule formation in the medulla. These inflammatory changes of follicular hyperplasia and macrophage infiltration may increase glucose uptake. Similarly, our findings suggest that LNs with CHA are likely benign, whereas those with a longer short-axis dimension or higher [^18^F]FDG SUVmax tend to be malignant. Based on these 4 risk factors (high LN risk category, large LN short-axis size (≥ 1.0 cm), absence of LN CHA and higher LN FDG SUV_max_ (≥ 10.1)), we grouped LNs according to their metastatic potential. The coexistence of 4 risk factors was an excellent predictor of LN metastases (PPV 100%). The [^18^F]FDG negative group can effectively predict benign lesions (NPV 95.0%). Hence, evaluation of these LNs requires only an [^18^F]FDG PET/CT examination. However, the PPV for LNs with 1–3 risk factors was 21.7%, suggesting that the nature of these LNs remains ambiguous and may necessitate additional testing.

Several articles evaluated the role of FAPI PET/CT in lung cancer and suggested that FAPI was a promising tumor imaging agent for lung cancer [11–13]. Similar to previous studies [13], [^18^F]FAPI demonstrated high specificity in this study, and the specificity of [^18^F]FAPI was higher than that of [^18^F]FDG. The sensitivity of FAPI for detecting LN metastases (80%) in this study was in accordance with the literature (84%) [13]. Previous studies reported that the SUV_max_ of FAPI was significantly higher than that of FDG in metastatic LNs [11–13]. In our study, however, there was no difference between [^18^F]FAPI and [^18^F]FDG SUV_max_ in metastatic LNs. This may be caused by that larger lymph nodes (≥ 1.0 cm) had higher FAPI uptake than smaller LNs. The majority of patients had advanced lung cancer with larger LN metastases in prior studies [11–13]. However, the proportion of larger LNs (18.2%) in our study was relatively low. The AUC of [^18^F]FAPI PET/CT for detecting LNs metastases was higher than that of [^18^F]FDG PET/CT (0.67), indicating the better predictive value of [^18^F]FAPI. In addition, FAPI uptake was found to be an independent predictor for LN metastases in multivariable analysis. Finally, [^18^F]FAPI is more accurate than [^18^F]FDG in diagnosing LNs (*P* < 0.001).

Indeed, [^18^F]FAPI contributed additional valuable information after [^18^F]FDG PET/CT. In stage I-IIIA NSCLC, our study highlighted the utility of [^18^F]FAPI PET in cases of an inconclusive diagnosis after [^18^F]FDG PET/CT examination, such as LNs with 1–3 risk factors. Under this circumstance, LNs with a FAPI SUV_max_ of less than 6.2 were considered as benign (NPV 93.8%). Conversely, LNs with a FAPI SUV_max_ ≥6.2 and without CHA were identified as metastatic (PPV 87.5%). Our research helps to resolve the ambiguous diagnosis of the LN nature in stage I-IIIA NSCLC. Our study proposes a complete diagnostic flowchart that may allow better identification of the LN nature in clinical practice (Fig. 9).

Due to a high uptake of [^18^F]FDG in inflamed LNs in the mediastinum and bilateral hilum [20, 21], misdiagnosis of LN metastasis and the overestimation of N staging are frequently observed. The clinical consensus recommends that positive mediastinal LNs on [^18^F]FDG PET/CT should be histologically verified via fine-needle aspiration biopsy [22]. However, the invasiveness of this procedure is a notable drawback. Moreover, radical surgery cannot proceed if a misdiagnosis of N3 stage occurs. Our study found that most of these incorrect N stages could be corrected with [^18^F]FAPI. After integrating [^18^F]FDG and [^18^F]FAPI PET/CT, the overestimation of the N stage decreased dramatically, from 58.5% (FDG-based) to 3.8%. The accuracy of [^18^F]FDG and [^18^F]FAPI PET/CT combined was as high as 83.0%. Thus, the number of patients requiring LN biopsies was significantly reduced. Finally, 18 patients initially staged as N3 based on [^18^F]FDG imaging were corrected to N0-N2 stage after the combination of [^18^F]FDG and [^18^F]FAPI PET/CT, ultimately enabling them to undergo radical surgery.

Currently, no imaging test exists that can detect LN metastasis with high sensitivity and specificity. Therefore, anatomical lobectomy, along with systematic LN dissection, is considered to be the main surgical approach for early-stage NSCLC [23]. Nevertheless, for patients without LN metastasis, systematic LN dissection results in excessive LN dissection. Consequently, this approach extends the surgery duration and increases perioperative complications. In this study, compared to [^18^F]FDG and [^18^F]FAPI PET/CT separately, the combination of [^18^F]FDG and [^18^F]FAPI PET/CT exhibited the highest detection accuracy for N staging, particularly in patients with pathological N0 stage. For pathological N0 stage patients, FDG-based N staging was overestimated in 25 patients, whereas only one patient was overestimated after integrating [^18^F]FDG and [^18^F]FAPI PET/CT. Out of these 25 FDG-positive patients, 24 patients were correctly identified as N0 stage through the combination of [^18^F]FDG and [^18^F]FAPI PET/CT. Therefore, these 24 patients did not need LN dissection during surgery, thereby reducing the burden on patients and shortening the length and difficulty of the operation. Our study emphasizes that if preoperative [^18^F]FDG imaging fails to clarify the nature of LNs, further [^18^F]FAPI could contribute to identifying LN nature and making more precise clinical decisions.

Our study has several limitations. First, the relatively small number of participants and the small proportion of patients with LN metastases may lead to statistical uncertainty. Second, the lack of immunohistochemical staining of FAP prevented us from clarifying the histological cause of the inconformity between FAPI PET/CT and pathology. Third, the limitations include the study design itself, since negative nodes were never sampled, which could introduce bias to the sensitivity, specificity, PPV and NPV estimates.

## Conclusion

Both [^18^F]FDG and [^18^F]FAPI PET/CT are valuable in diagnosing of LNs in patients with stage I-IIIA NSCLC. When the nature of the LNs is ambiguous with [^18^F]FDG PET/CT alone, the SUV_max_ of LNs by [^18^F]FAPI can provide essential information. The combination of [^18^F]FDG and [^18^F]FAPI PET/CT yielded the highest detection accuracy for N staging, thus facilitating more precise clinical decision-making.

## Data Availability

The datasets used and analysed during the current study are available from the corresponding author on reasonable request.

## Abbreviations

[^18^F]: Fluorine-18
[^68^Ga]: Gallium 68
CHA: Calcification or high-attenuation
FAPI: Fibroblast activation protein inhibitor
FDG: Fluorodeoxyglucose
IASLC: International Association for the Study of Lung Cancer
LN: Lymph node
NPV: Negative Predictive Value
NSCLC: Non-small cell lung cancer
PET/CT: Positron-emission tomography/computed tomography
PPV: Positive Predictive Value
SUV_max_: The maximum standard uptake value
